# Parents’ Perceptions of Pediatric Surgical Patients Regarding Animal-Assisted Therapy: A Qualitative Study

**DOI:** 10.3390/healthcare13172207

**Published:** 2025-09-03

**Authors:** Felice Curcio, Dhurata Ivziku, Simona Pirisinu, Luca Bertocchi, Giovanni Gioiello, Francesco Saverio Camoglio, Adalberto Rangel Restrepo, Ippolito Notarnicola, Cesar Ivan Aviles Gonzalez

**Affiliations:** 1 Faculty of Medicine and Surgery, University of Sassari (UNISS), Viale San Pietro 43/B, 07100 Sassari, Italy; felice.curcio@aouss.it (F.C.); simonapirisinu@gmail.com (S.P.); 2 Department of Health Professions, Fondazione Policlinico Universitario Campus Bio-Medico, 00128 Rome, Italy; d.ivziku@policlinicocampus.it; 3 Hematology Unit, Trieste University Hospital (ASUGI), Piazza dell’Ospitale 1, 34129 Trieste, Italy; 4 Department of Medicine and Surgery, University of Enna “Kore”, Piazza dell’Università, 94100 Enna, Italy; ippolito.notarnicola@unikore.it (I.N.);; 5 Department of Medicine, Surgery and Pharmacy, Sassari University, Viale San Pietro 43/B, 07100 Sassari, Italy; fscamoglio@uniss.it; 6 Faculty of Administrative, Accounting and Economic Sciences, Universidad Popular del Cesar, Valledupar 200002, Colombia; adalbertorangel@unicesar.edu.co

**Keywords:** Animal-Assisted Therapy, child health, complementary therapies, qualitative research

## Abstract

**Background/Objectives**: Pet therapy, also known as Animal-Assisted Therapy (AAT), is increasingly recognized for its potential to support pediatric patients and their families in the hospital setting. This study explores the perceptions of parents of pediatric surgical patients regarding the use of pet therapy during hospitalization. **Methods**: A qualitative study was conducted using Interpretative Phenomenological Analysis (IPA). Semi-structured interviews were conducted with parents of children admitted for surgery procedures. The interviews were transcribed, read thoroughly, and analyzed. Thematic analysis was employed to identify recurring themes related to the emotional, relational, and organizational aspects of pet therapy. **Results**: Twenty mothers were interviewed and five main themes emerged: (1) *general perception of pet therapy* (valuable tool to reduce children’s anxiety and provide emotional support); (2) *parental expectations about benefits for their children* (positive interactions between children, parents and caregivers); (3*) expected behavioral impact on the child* (animals were viewed as mediators of relational bonding, especially in stressful moments); (4) *emotional repercussions on caregivers* (parental well-being improved when children appeared calmer during sessions); and (5) *preferences regarding animals and organizational aspects* (most parents preferred dogs for their empathic and communicative nature, while horses were appreciated but considered impractical in a hospital setting). In addition, the presence of structured AAT programs also positively influenced parents’ perception of the quality of care. Nurses played a crucial role in supporting the implementation of AAT and family involvement, aligning with the Family-Centered Care model. **Conclusions**: This study found that parents view AAT as a valuable intervention that reduces anxiety and supports emotional well-being in hospitalized children. Nurses play a vital role in integrating AAT within Family-Centered Care to enhance the pediatric hospital experience.

## 1. Introduction

In recent years, pet therapy, also known as Animal-Assisted Therapy (AAT), has been the subject of growing attention as a possible complementary approach in healthcare, particularly in hospital settings [1]. AAT is a structured, goal-oriented intervention in which a specially trained animal collaborates with a qualified professional to promote the achievement of personalized therapeutic outcomes. Activities may include physical interaction, play, socialization exercises, sensory stimulation, and emotional support, with the animal serving as a relational and motivational mediator [1,2]. The therapeutic value of human–animal interactions has long been acknowledged, with historical and contemporary evidence highlighting their positive effects on emotional well-being and stress reduction [2].

Recent systematic reviews have shown the efficacy of AAT in reducing fear, anxiety, and stress in children and adolescents patient through both physiological and subjective mechanisms [3,4,5]. These effects are particularly relevant in pediatrics, where hospitalization is often a cause of discomfort and distress for children and families [1,6,7,8].

The emotional state of parents plays a crucial role in shaping the child’s coping strategies during hospitalization and surgery. When parents are able to regulate their own distress and adopt positive coping mechanisms, children display greater emotional regulation and resilience, whereas parental anxiety or avoidance tends to intensify the child’s distress [9]. In this sense, interventions such as AAT may exert systemic effects, simultaneously supporting children and caregivers [10].

The majority of studies and clinical experiences highlight dogs as the preferred animal in therapeutic settings. Some specific characteristics such as their cognitive and emotional capacities and biases, their evolutionary connection with humans, as well as a natural attraction and emotional connection between both species make dogs particularly suitable for AAT [11,12].

Recent randomized trials have demonstrated that even small animals, such as goldfish and aquatic turtles, can effectively reduce pain, anxiety, and fear during invasive procedures like iv catheter insertion, further supporting the potential of AAT in pediatric clinical practice [6].

Beyond these animals, horses have also been considered in AAT. Although less common in hospital wards due to practical constraints, equine-assisted therapy has shown benefits for children with neurological or motor disabilities and, when applied in rehabilitation or outdoor settings, can enrich and diversify hospital services [13,14].

For AAT to be effectively integrated into pediatric hospital wards, however, a structured planning process is required, along with the definition of specific prerequisites and organizational requirements. The literature emphasizes that ensuring safety, promoting multidisciplinary collaboration, and carefully selecting both patients and animals are essential conditions for the successful and sustainable implementation of AAT in healthcare contexts, especially in highly sensitive areas [15].

In the context of pediatric surgery, AAT has shown auspicious outcomes. Sobo et al. [16] provided one of the first pieces of evidence that pet therapy, particularly visits by trained dogs, can significantly reduce perceived pain in pediatric patients during the postoperative period, likely through cognitive distraction mechanisms, suggesting its potential as a valuable complement in pediatric pain management. Similarly, Calcaterra et al. [17] observed that children who interacted with therapy dogs after surgery demonstrated improved emotional regulation and reduced pain perception. Ávila-Álvarez et al. [18] also confirmed that even short AAT sessions can enhance children’s mood and increase parents’ satisfaction with hospital care.

Nurses play a pivotal role in implementing pet therapy interventions, as they maintain constant, often 24-h, contact with both patients and their families throughout hospitalization [6,7,8]. Indeed, pet therapy is identified under code 4320 in the Nursing Interventions Classification (NIC) system, reflecting its integration into standard nursing care practices [19]. The NIC, particularly when integrated with NANDA-International diagnoses and the Nursing Outcomes Classification (NNN linkages), is one of the most widely used standardized nursing terminologies recognized by the American Nurses Association [20,21]. It defines and categorizes nursing interventions, supports evidence-based practice and interdisciplinary communication, facilitates integration into individualized care plans, and ultimately enhances patient outcomes [20,22]. The inclusion of pet therapy in this classification reflects its relevance in holistic, person-centered, and outcomes-driven nursing care. This approach aligns with the principles of the Family-Centered Care (FCC) model, which emphasizes the active involvement of families in all aspects of the care process [23].

The FCC model is rooted in an authentic and profound collaboration between families and healthcare professionals, where the family is welcomed as an essential part of the care team. This approach not only recognizes but also respects and values the cultural beliefs, unique needs, and emotions of each family. In doing so, it nurtures a relationship built on trust, empathy, and shared responsibility, transforming care into a journey that is not only personalized, but also profoundly human, compassionate, and capable of embracing the emotions that accompany the care experience [24,25,26].

In pediatric settings, where emotional security and relational continuity are vital, AAT can serve as a relational tool that strengthens the bond between children, families, and healthcare professionals [23,27].

Within this approach, nurses’ involvement is crucial to ensure safe and therapeutic interactions, as well as to support the emotional and physical benefits of AAT [1]. Moreover, London et al. [28] emphasized the importance of parental perspectives in evaluating therapeutic outcomes. Their qualitative study on AAT for children with autism spectrum disorder highlighted improvements, according to caregivers, in communication, behavior regulation, and social participation.

Despite the growing body of scientific evidence, qualitative research focusing specifically on parents’ perspectives of AAT in pediatric surgical settings remains limited. This study aims to fill this gap by exploring the perception of parents whose children have undergone surgery, regardless of whether the children actually participated in AAT programs. The aim is to gain a deeper understanding of how parents perceive the role of pet therapy in their children’s surgical experience and perceived well-being.

## 2. Materials and Methods

### 2.1. Study Design

This study employed Interpretative Phenomenological Analysis (IPA) as a qualitative research methodology to explore the perceptions of parents of pediatric surgical patients regarding pet therapy. This approach was selected for its capacity to deeply investigate participants’ lived experiences, allowing for an in-depth understanding of their interpretations, beliefs, and perspectives through a phenomenological lens focused on the meanings individuals ascribe to specific phenomena.

To ensure methodological rigor and clear, structured reporting of results, the study adhered to the Consolidated Criteria for Reporting Qualitative Research (COREQ) guidelines [29].

### 2.2. Sampling and Recruitment

Between February and March 2025, participants were recruited using convenience sampling from the Pediatric and Neonatal Surgery Unit at the University Hospital of Sassari (Italy). Recruitment continued until data saturation was reached. No recruited individuals refused to participate, and each participant was assigned an alphanumeric code to ensure anonymity.

Eligible participants met the following inclusion criteria: (1) being of legal age; (2) being the mother of a child aged between 1 and 16 years; (3) willingness to provide written informed consent. Exclusion criteria included: (1) not being a native Italian speaker; (2) being the mother of a child undergoing urgent/emergency surgery.

### 2.3. Data Collection

Participants were interviewed on the day of their child’s surgery. Interviews were conducted in a dedicated patient room to ensure participant privacy and confidentiality. None of the participants declined the interview.

The interviews were conducted by SP, who received specific training through a workshop on qualitative interviews and conducted several pilot interviews prior to the study to gain practical experience. The interview process was supervised and guided by FC, a pediatric nurse with approximately 15 years of clinical experience, an expert in phenomenological analysis, and co-author of numerous qualitative studies. In addition, a standardized interview guide was used to ensure consistency in the key questions across all participants.

Prior to data collection, the researchers performed bracketing by recording their assumptions, beliefs, and preconceived ideas regarding the phenomenon under investigation. By applying this reflective technique before data collection and analysis, researchers aimed to minimize potential bias and enhance objectivity in the interpretive process [30].

Data were collected through in-depth, face-to-face, semi-structured individual interviews. Semi-structured interviews are the most commonly used method in IPA studies, as they elicit rich, first-person accounts of participants’ experiences with the phenomenon of interest. This format provides a consistent framework for exploring specific topics while allowing for the collection of rich, reliable, and comparable qualitative data [31].

An interview guide (Appendix A) was used flexibly to support a natural flow of conversation and to encourage participants to elaborate on their perceptions while systematically exploring various aspects of the main topic. The initial question served as an entry point to introduce the topic. Interviews lasted between 20 and 30 min, were audio-recorded, and the recordings were transcribed verbatim for analysis. In addition to the interviews, participants completed a short questionnaire to collect sociodemographic information essential for analysis and for describing the general characteristics of the study sample.

### 2.4. Data Analysis

Data analysis was conducted by the principles of IPA, as outlined by Smith et al. [32]. The identification of themes followed a primarily inductive approach, allowing themes to emerge from participants’ narratives rather than from predefined theoretical constructs. At the same time, the process was interpretive, recognizing the active role of researchers in making sense of participants’ narratives.

All interviews were listened to multiple times and transcribed verbatim. Each transcript was read and reread to allow researchers to become fully immersed in the content. Subsequently, two researchers (FC and GG) independently conducted a detailed analysis of each transcript. Descriptive, linguistic, and conceptual comments were annotated in the left-hand margin, while emerging themes were recorded in the right-hand margin, reflecting both the transcript and the initial notes. These emergent themes were then interrelated to construct a coherent and organized thematic account of the phenomenon. Multiple connections were established, resulting in the development of a set of superordinate themes that spanned the entire dataset.

### 2.5. Rigour

To minimize the influence of researchers’ preconceptions during category extraction, the process of bracketing was initially undertaken. This technique, described by Cohen et al. [33], involves critical reflection aimed at ensuring analytical rigor.

The trustworthiness of this study was ensured through adherence to high standards of dependability, confirmability, credibility, and transferability, as proposed by Streubert and Carpenter [34]. Rigorous and precise methods were employed at every stage of the inquiry—from data collection to analysis and interpretation, including peer review procedures to minimize interpretative errors. The peer review process was conducted by two independent researchers who were not directly involved in data collection. Each researcher independently reviewed the coding framework and thematic categorization, and discrepancies were resolved through discussion until consensus was reached. This procedure minimized the risk of interpretation bias.

Interview data were transcribed, incorporating participants’ direct quotations, and analyzed through a thematic approach, thereby safeguarding the authenticity of the participants’ perspectives. Clear inclusion/exclusion criteria, contextual descriptions, participant characteristics, and detailed procedures for data collection and analysis were provided. To reduce confirmation bias, findings were discussed among the research team and shared with participants for validation. In particular, member checking was performed, meaning that all interviewees were invited to review and confirm the results, allowing for critical reflection and increasing the accuracy of interpretations. These measures enhance the validity, relevance, and rigor of the findings, contributing to a comprehensive understanding of the phenomenon under investigation.

### 2.6. Ethical Considerations

This study was conducted in accordance with the ethical guidelines outlined in the Declaration of Helsinki [35], the Italian Privacy Law (Legislative Decree No. 196/2003), and the EU General Data Protection Regulation (GDPR 2016/679). Ethical approval was obtained from the hospital’s director.

All participants were fully informed, both verbally and in writing, about the purpose of the study. Prior to the interviews, each participant signed an informed consent form, acknowledging their understanding of the study objectives, the voluntary nature of their participation, and their right to withdraw at any time without consequence.

To ensure privacy, all data were anonymized using alphanumeric codes and securely stored in encrypted files accessible only to the research team.

## 3. Results

### 3.1. Participant Characteristics

The sample included 20 mothers of children scheduled for elective surgery. The mean age of participants was 39.6 years (SD ± 6.98). Most participants had completed secondary education. Table 1 provides a detailed overview of the demographic characteristics of the participants.

### 3.2. Emerging Themes

The qualitative analysis of the interviews identified five main themes that represent the experiences and perceptions of parents regarding the introduction of pet therapy in pediatric surgical hospital settings:

1. General perception of pet therapy
2. Parental expectations about benefits for their children
3. Expected behavioral impact on the child
4. Emotional repercussions on caregivers
5. Preferences regarding animals and organizational aspects

#### 3.2.1. Theme 1: General Perception of Pet Therapy

Across all interviews, parents expressed a largely positive view of pet therapy. This approach was frequently described as an emotional support tool capable of providing relief to children during vulnerable moments such as hospitalization and surgery. The presence of an animal was associated with a calming and reassuring effect, helping to distract from fear and reduce anxiety:

*“It is a form of support provided by the animal to relieve anxiety, fears, or worries”,*

*(P_1)*

*“It helps to emotionally and even physically support the child’s well-being”.*

*(P_3)*

Several parents emphasized that the presence of an animal, especially before admission, could make the hospital environment feel more welcoming and less impersonal. Pet therapy was seen as a means to transform the hospital setting into a more emotionally responsive and familiar space for the child: *“A space that is not just hospital-like, but closer to you” (P_5).*

Even among parents with limited prior knowledge of pet therapy, interest and openness were evident:

*“I do not know how to describe it well, but I think it is a good thing”,*

*(P_7)*

*“I had not heard much about it, but I think it is useful. Animals help, they keep you company, they distract you”.*

*(P_11)*

The affective and empathetic nature of animals emerged repeatedly in parents’ narratives. Animals—especially dogs—were seen as capable of establishing deep emotional bonds with people and responding sensitively to human emotions. Some parents used expressions that highlighted this belief:

*“To me, a dog is a human being on four legs”,*

*(P_14)*

*“Sometimes, they understand you better than people do”.*

*(P_17)*

#### 3.2.2. Theme 2: Parental Expectations About Benefits for Their Children

Many parents acknowledged AAT as a valuable support in managing preoperative stress, attributing to it the ability to distract and calm the child during the most critical phases of hospitalization.

Interaction with the animal was perceived as an opportunity to shift attention away from anxiety-provoking thoughts associated with surgery and the clinical environment, thus reducing emotional tension*: “To distract and calm him down… to take his mind off what he is about to undergo.” (I_1).* In this sense, the presence of the animal not only fosters a more serene approach to the hospital experience but also improves the child’s willingness to undergo medical procedures: *“It would relax him. It would help him experience the surgery better.” (I_16).*

In addition to the immediate benefits related to distraction and relaxation, some parents highlighted a positive impact on social and relational dynamics, viewing the animal as a stimulus for openness and communication: *“It would redirect his thoughts and help him open up, even socially.” (I_20).* This underscores the added value of AAT in supporting the child not only in coping with the hospital environment but also in reinforcing social skills in a delicate context.

Finally, there emerged the perception that AAT might be more effective than traditional distraction strategies such as digital games, given its more tangible and emotionally engaging nature: *“It would be better than giving him a game… more real, closer to his world.” (I_5).* This observation reinforces the idea that the animal can serve as an authentic bridge between the child and the hospital setting, making the experience more human and less alienating.

#### 3.2.3. Theme 3: Expected Behavioral Impact on the Child

Parents attributed profoundly beneficial effects to the interaction with animals, particularly in children experiencing emotional distress, agitation, or hyperactivity. Animals were perceived as capable of inducing a greater state of calm and serenity, even in children who are particularly active or emotionally fragile: *“He would be calmer, more serene. Animals can do that.” (I_16)* and *“My son is very energetic, but if there is a dog near him, he immediately settles down.” (I_14).*

The emotional bond created with the animal was, in some cases, described as intense enough to trigger immediate reassurance and emotional stabilization: *“My daughter calms down as soon as she sees an animal—she adores dogs.” (I_19).*

Beyond the direct benefits for the child, AAT was also seen as a valuable tool to improve interaction with healthcare staff. Some parents emphasized how the presence of an animal could break down relational barriers and help make children more receptive to physical contact and care: *“The dog helps to break barriers… it calms them so they can be touched.” (I_6).* This suggests the mediating potential of AAT, extending beyond emotional comfort to impact the broader therapeutic context.

However, one parent expressed caution, raising doubts about the intervention’s effectiveness in the presence of neurodevelopmental disorders and highlighting the variability in individual responses: *“My son is autistic, maybe he would have reacted differently—I am not sure it would have helped him.” (I_15).*

#### 3.2.4. Theme 4: Emotional Repercussions on Caregivers

The perceived well-being of the child directly influenced the parent’s emotional balance, creating a strong link between the child’s emotional state and that of the caregiver. Several parents highlighted how their child’s serenity brought them a sense of relief and personal tranquility: *“If he is calm, I am calm. Seeing him smile reassures me.” (I_11).* In this context, AAT was not experienced solely as a tool to support the child, but also as an indirect resource for the parent, capable of providing emotional comfort and psychological support.

Some parents recognized in animals a form of shared companionship, capable of alleviating anxiety and reducing feelings of helplessness, as expressed by one participant: *“It would help me manage anxiety, like companionship—for both of us.” (I_20).* Even in moments of physical absence, the knowledge that the child could benefit from the presence of an animal served as a source of reassurance: *“When I cannot be there, knowing he has support makes me feel better.” (I_5).*

Furthermore, the role of the animal was described not only in terms of individual benefit but also as a potential facilitator of relationships within the family unit. Some parents emphasized the relational value of the animal, offering a form of consolation that complements and enhances parental care: *“It is a different kind of comfort than what I can give as a parent.” (I_4).*

#### 3.2.5. Theme 5: Preferences Regarding Animals and Organizational Aspects

Almost all parents identified the dog as the most suitable animal for AAT, due to its ability to establish a genuine, empathetic, and communicative relationship with the child. The dog’s sensitivity and the perception of a kind of “humanity” in its behavior make it, in the eyes of many parents, an ideal companion in therapeutic settings: *“The dog is the only animal that truly has human feelings.” (I_19).* However, a degree of flexibility also emerged regarding the choice of animal, depending on the child’s specific preferences and needs.

Some parents expressed openness to other species such as rabbits or fish, recognizing their potential calming or emotional benefits: *“Even a little bunny would be fine, if the child likes it.” (I_18)*, and *“A fish in the room would calm him down—he already has one at home.” (I_17).*

Another theme that emerged from the interviews was the reference to the horse as a therapeutic animal. Despite parents’ awareness of the logistical challenges associated with bringing a horse into a hospital setting, it was described as a highly empathetic animal capable of forming deep emotional bonds and making a significant contribution to children’s emotional well-being. As one parent put it: *“The horse connects on a deep level with the person.” (I_3),* and *“The horse is very respectful.” (I_6).* Nonetheless, it was recognized that, for structural and organizational reasons, the presence of a horse is more appropriate in outdoor contexts or specific rehabilitation programs rather than within a hospital ward: *“It would be the dog, because a horse in the hospital would not work.” (I_1).*

Regarding safety and hygiene, most parents expressed confidence in the hospital’s procedures, showing little concern or resistance. The prevailing perception was that the presence of animals in the hospital is subject to rigorous protocols that ensure appropriateness and safety: *“I assume that if it is allowed in the hospital, it must be safe.” (I_7).* In some cases, greater concern was expressed about people than animals themselves: *“I worry more about people than about animals.” (I_6).*

Finally, many participants considered the availability of AAT a significant factor in choosing a healthcare facility, to the point of becoming a decisive criterion when the quality of care was otherwise equal: *“I would choose the one with pet therapy, no doubt.” (I_17),* and *“If the healthcare teams were equal, I would choose the one with pet therapy.” (I_1, I_5, I_9).*

## 4. Discussion

This study aimed to explore the perceptions of parents of pediatric surgical patients regarding the introduction of AAT in hospital settings. The findings reveal a broadly positive perception of AAT among parents, who describe it as a valuable source of emotional support and an effective tool for distracting children during hospitalization.

Regarding the first emergent theme, the presence of an animal is perceived as reassuring and capable of diverting the child’s attention from fears and concerns related to surgery and the hospital environment. Interviewed parents emphasized that AAT can transform anxiety-filled waiting moments into experiences of serenity, helping to make the hospital setting more humane and welcoming.

AAT in hospitals is recognized as an effective intervention for reducing anxiety and enhancing emotional well-being in both children and adults. Recent studies have shown that even brief encounters with animals lead to significant reductions in anxiety among pediatric patients, with high satisfaction levels also reported among parents, who perceive the hospital environment as more humane and comforting thanks to these activities [1,2,36]. Our findings confirm this evidence, but also significantly expand upon it. While most of the existing literature has focused on quantifiable outcomes, such as reductions in anxiety scores, our qualitative approach highlights the interpretative dimension, revealing how parents themselves understand these benefits. Specifically, parents described AAT not only as a distraction tool, but as an intervention that contributes to the humanization of care and the strengthening of family resilience. This perspective suggests that the significance of AAT may go beyond momentary distraction, contributing to broader psychosocial well-being for both child and family.

A particularly relevant finding concerns the perception of animals as relational mediators, capable of facilitating communication between the child and healthcare professionals. This element emerged strongly from the interviews and is supported by evidence from Vagnoli et al. [37], which demonstrates that the presence of animals encourages greater acceptance of medical procedures, such as venipuncture.

Another key theme was the expectation that AAT might serve as a healthier and more developmental alternative to traditional forms of entertainment, such as electronic devices. Several parents noted that interaction with animals supports emotional and relational development in children, going beyond mere distraction. These insights are consistent with the scientific literature, which confirms the effectiveness of AAT in reducing preoperative anxiety and promoting more adaptive coping strategies among hospitalized children [2,36]. What our study adds is the emphasis parents placed on AAT as an active, socially and developmentally constructive experience, explicitly contrasted with the passivity often associated with electronic entertainment—pointing to educational value in addition to clinical benefit.

Parents described a positive anticipation of the animal’s ability to instill calm and tranquility in their children, thus facilitating their adaptation to the hospital environment and medical procedures. However, it should be noted that some children in our sample presented additional diagnoses beyond their surgical condition, including neurodevelopmental disorders. Parents themselves acknowledged that responses to AAT may vary significantly in these populations, as also highlighted by several authors [28,38,39], who emphasize the need for tailored interventions. This heterogeneity indicates that parental experiences should not be interpreted as uniform, and particular caution is warranted when considering the effectiveness of AAT in children with neurological or neurodevelopmental conditions. Nevertheless, as Beetz et al. [40] report, animals can support emotional and behavioral regulation in children, especially those with heightened reactivity or psychological vulnerability. Therefore, although responses to AAT may differ depending on individual characteristics—particularly in children with neurodevelopmental disorders—the therapeutic potential of such interventions remains substantial [41]. Furthermore, the presence of an animal, along with the parent’s positive perception of it, may contribute to a sense of safety and predictability—elements particularly valuable for children with difficulties in emotional or behavioral regulation. From this perspective, a need emerges to design AAT protocols that consider not only the type of disorder but also the child’s temperament and personal preferences, as well as those of the parent. This personalized approach could significantly enhance the efficacy of interventions, fostering greater engagement and improving both physiological and psychological outcomes for the entire family.

The fourth theme emerging from this study concerns the impact of AAT on parental well-being. Many participants reported that seeing their child calmer and more relaxed, thanks to the presence of the animal, helped them better manage the anxiety, worry, and sense of helplessness typically associated with the hospital environment. This is particularly relevant because parental emotional state is a key determinant of the child’s coping during hospitalization. When parents implement positive dyadic coping strategies, especially those related to emotion regulation, children show more effective emotional regulation and a higher quality of life, even in the presence of chronic conditions or family stress [42,43]. Conversely, negative or avoidant coping strategies increase the risk of depressive symptoms, behavioral problems, and adjustment difficulties [44]. Accordingly, AAT benefits appear to propagate beyond the individual child, producing systemic effects on the family unit. Framing AAT as a family-centered strategy highlights its potential to reduce caregiver burden and to foster more positive parent–child dynamics during the perioperative trajectory. This study reveals the perceived role of the animal as a relational mediator, capable of strengthening the parent–child bond and creating moments of shared comfort and emotional closeness, thereby transforming a challenging experience into an opportunity for mutual support. Existing literature confirms these findings: parents who take part in AAT programs report reductions in anxiety and stress, with beneficial effects also observed in high-complexity settings such as pediatric oncology and intensive care units [45,46,47].

The fifth and final theme concerns preferences regarding animals and organizational aspects. The findings show a marked parental preference for dogs as the ideal animals for AAT, with 70% identifying them as the most suitable for pet therapy. This emphasizes their unconditional affection and capacity to establish genuine relationships with children. This aligns with the literature, which recognizes the dog’s strong empathic, communicative, and social abilities, particularly suited for therapeutic interaction with children [33]. Our findings suggest that this preference is also shaped by cultural representations of dogs as reliable companions and protectors, which may increase parental trust and perceived safety of the intervention [11].

Meanwhile, 25% of parents considered the horse to be the most suitable animal for AAT. According to the interviews, horses were valued for their relational and empathic qualities, consistent with the literature on the effectiveness of hippotherapy, especially for children with motor or neurological disabilities [48]. An original insight that emerged was the cultural and symbolic value attributed to the horse, particularly in the Sardinian context. Some parents noted that the choice of horse was not solely based on therapeutic criteria but also reflected a strong connection with local traditions and territory. This perspective aligns with Peterson [49], who emphasizes that human–animal relationships are profoundly shaped by sociocultural contexts. However, parents also acknowledged the practical limitations of using horses in hospital settings, viewing them as more appropriate for outdoor environments or specific rehabilitation programs. In support of this observation, a study by Bisht and colleagues [50] highlighted that the extension of AAT programs to other animals, such as horses, poses significant safety challenges. The study indeed showed that horses can carry human opportunistic pathogens (Acinetobacter baumannii, E. coli, Pseudomonas aeruginosa), thus recommending strict infection prevention protocols and a limitation of contact points during hospital visits.

From an organizational perspective, parents reported trust in hygiene and safety protocols, in line with international recommendations [37]. The presence of a structured AAT program was also perceived as a distinctive element in the choice of the healthcare facility, with direct effects on both user satisfaction and the hospital’s reputation. Although empirical evidence supporting the role of pet therapy as a hospital selection criterion is not yet solid, some studies show a growing interest in its introduction into clinical settings. In particular, Reddekop et al. [51] found that about 80% of emergency room patients would welcome a visit from a therapy dog, while Hinic et al. [36] reported that all parents interviewed (*n* = 46; 100%), after participating in a pet therapy session, would appreciate another visit in the event of a new hospitalization and would recommend the experience to others. Embedding AAT within formal care pathways may thus function as both a clinical and organizational innovation—strengthening family engagement while differentiating services in competitive healthcare systems.

These findings confirm and expand upon existing evidence regarding the perceived value of AAT in pediatric hospital settings, underlining the importance of such complementary interventions in promoting family well-being [1,2].

### Nursing Implications and Family-Centered Care

AAT emerges not only as a tool to promote the child’s well-being but also as a relational and psychological resource for caregivers, contributing to an overall improvement in the hospitalization experience. This aligns with the principles of Family-Centered Care [27,52]. Within this framework, the involvement of nursing professionals is strategically guided through the use of standardized nursing classifications such as the NIC. Although NIC was not applied in this study, standardized nursing terminologies, when integrated with NANDA-International diagnoses and NOC (NNN linkages), can facilitate systematic planning, implementation, and evaluation of interventions, enhance communication and support measurable outcomes [20,21,22]. In addition to the specific intervention code 4320 (Pet Therapy), other complementary nursing activities play a crucial role in supporting the effectiveness of AAT and enhancing family engagement.

Among the nursing interventions that support AAT are emotional support—helping both the child and family manage their emotions and fostering a positive interaction with the animal—and promoting communication between the family and healthcare team, encouraging an open and collaborative dialogue. Nurses also contribute to reducing anxiety in both the child and caregivers by employing AAT as a complementary therapeutic resource.

The Family-Centered Care model encourages the active participation of the family in all stages of the care process, recognizing them as integral members of the healthcare team [53]. In this sense, AAT is an effective strategy for fostering a positive and collaborative relational climate.

In particular, hospital visitation programs that allow patients to meet with pets, aimed at preserving the human–animal bond, have been shown to stimulate compassion, connection, and reciprocity among patients, providers, and families. These encounters favor meaningful conversations, healing relationships, and deeper understanding of the patient’s psychosocial context, thereby contributing to more personalized care and overall well-being [54].

A positive perception of AAT among the families of hospitalized children awaiting surgery, when integrated into the Family-Centered Care approach, may foster greater family engagement and contribute to improving the quality of pediatric care. This model has the potential to enhance patient and family satisfaction, reduce anxiety and stress, support psychological well-being, and, in some cases, positively impact both the costs and the length of hospitalization [25,55].

This approach more effectively addresses the biopsychosocial needs of young patients and their caregivers.

Given their continuous presence and educational role, nurses are key figures in integrating AAT safely and coherently into the care plan. They are responsible not only for providing accurate information and ensuring clinical safety but also for monitoring the emotional, relational, and cultural dimensions that characterize each family unit.

## 5. Conclusions

This study highlights that parents perceive AAT as a valuable support during pediatric hospitalization, which helps reduce children’s anxiety and enhances their emotional well-being. Animals, especially dogs, serve as effective relational mediators, facilitating communication between children and healthcare staff while providing comfort and distraction. Parents view AAT not only as entertainment but as a meaningful, developmentally supportive intervention that fosters family engagement. Tailoring AAT to individual needs, particularly for children with neurodevelopmental disorders, and considering cultural preferences is essential. Integrating AAT within a Family-Centered Care framework positions nurses as crucial facilitators in delivering safe, holistic care. Our findings emphasize that the benefits of AAT extend beyond the child, positively influencing parental well-being and reducing caregiver stress, thus producing systemic effects on the family unit. This broader impact highlights the potential of AAT as both a therapeutic and organizational innovation: on the one hand, it enhances clinical outcomes by supporting emotional regulation and resilience; on the other, it may contribute to hospital reputation and family satisfaction, positioning AAT as a factor that can influence the perceived quality of care. Finally, by confirming existing evidence while also providing new insights into parents’ subjective experiences, this study underscores the importance of designing personalized and culturally sensitive AAT protocols, capable of maximizing engagement and improving the overall pediatric care experience.

## Figures and Tables

**Table 1:** Characteristics of the sample (*n* = 20).

ID	Age	Education Level	Number of Children
1	36	University Degree	2
2	40	Lower Secondary School	3
3	46	Upper Secondary School Diploma	1
4	43	Upper Secondary School Diploma	2
5	36	University Degree	2
6	39	Upper Secondary School Diploma	1
7	45	Upper Secondary School Diploma	1
8	31	Lower Secondary School	2
9	28	Upper Secondary School Diploma	2
10	55	Upper Secondary School Diploma	1
11	46	Upper Secondary School Diploma	2
12	41	Upper Secondary School Diploma	2
13	46	Upper Secondary School Diploma	2
14	35	Lower Secondary School	2
15	35	University Degree	2
16	40	Upper Secondary School Diploma	2
17	41	Upper Secondary School Diploma	1
18	45	Upper Secondary School Diploma	2
19	25	Upper Secondary School Diploma	1
20	39	University Degree	1

## Data Availability

The data presented in this study are available from the corresponding author upon request.

## Appendix A. Interview Guide

1. Have you ever heard of “pet therapy”? If so, how would you describe it?
2. Have you ever had a personal experience with pet therapy, or do you know someone who has shared such an experience with you? Could you tell me about it?
3. Do you currently have any pets at home?
4. If yes, which kind? If not, could you please explain why you do not have any pets at home?
5. In your opinion, what are the potential positive effects of pet therapy? Are there any negative aspects?
6. Have you ever heard of pet therapy being implemented in hospitals or healthcare facilities?
7. What is your opinion about the possibility of including pet therapy as part of your child’s treatment plan?
8. What benefits should pet therapy offer your child in a hospital setting?
9. In general, how do you think a therapy animal might influence your child’s behavior during hospitalization?
10. Which animal do you consider most appropriate for pet therapy? Please explain your reasoning.
11. Do you have any concerns or reservations about pet therapy?
12. If you had to choose between two healthcare facilities for your child’s hospitalization or procedure, and only one of them offered a pet therapy program, would that affect your choice? Which one would you choose and why?
13. If pet therapy were to be offered in the ward where your child is admitted, would you allow your child to participate?
14. How do you think pet therapy could help you cope better with your child’s hospitalization?

## References

[1] Antolín Esteve L., López-Mases P., Bartolomé Del Pino L.E., Lázaro E. (2025). Animal-Assisted Therapy for the Management of Anxiety in the Hospital Setting: A Systematic Review. Holist. Nurs. Pract..

[2] Brandão C., Sampaio M., Sousa-Gomes V., Fávero M., Moreira D. (2025). Effects of Animal-Assisted Therapy for Anxiety Reduction in Children and Adolescents: A Systematic Review. J. Clin. Med..

[3] Fornefeld D., Zellin U., Schmidt P., Fricke O. (2025). The Supporting Role of Dogs in the Inpatient Setting: A Systematic Review of the Therapeutic Effects of Animal-Assisted Therapy with Dogs for Children and Adolescents in an Inpatient Setting. Eur. Child Adolesc. Psychiatry.

[4] Correale C., Borgi M., Collacchi B., Falamesca C., Gentile S., Vigevano F., Cappelletti S., Cirulli F. (2022). Improving the Emotional Distress and the Experience of Hospitalization in Children and Adolescent Patients Through Animal Assisted Interventions: A Systematic Review. Front. Psychol..

[5] Zhang Y., Yan F., Li S., Wang Y., Ma Y. (2021). Effectiveness of Animal-Assisted Therapy on Pain in Children: A Systematic Review and Meta-Analysis. Int. J. Nurs. Sci..

[6] Sarman A., Günay U. (2023). The Effects of Goldfish on Anxiety, Fear, Psychological and Emotional Well-Being of Hospitalized Children: A Randomized Controlled Study. J. Pediatr. Nurs..

[7] Sarman A., Tuncay S. (2024). Goldfish or Aquatic Turtle? Impact of Two Animal Assisted Interventions on Children’s Pain, Anxiety, and Fear during IV Catheterization: A Randomized Controlled Trial. J. Pediatr. Nurs..

[8] Sarman A., Can V., Tuncay S., Loutfy A. (2025). Determination of Nursing Students’ Opinions on the Use of Animal-Assisted Interventions in Pediatric Care and Their Animal Love Levels. Nurs. Health Sci..

[9] Ben-Ari A., Aloni R., Ben-David S., Benarroch F., Margalit D. (2021). Parental Psychological Flexibility as a Mediating Factor of Post-Traumatic Stress Disorder in Children after Hospitalization or Surgery. Int. J. Environ. Res. Public Health.

[10] Gaudet L.A., Elliott S.A., Ali S., Kammerer E., Stauffer B., Felkar B., Scott S.D., Dennett L., Hartling L. (2022). Pet Therapy in the Emergency Department and Ambulatory Care: A Systematic Review and Meta-Analysis. Acad. Emerg. Med..

[11] Bremhorst A., Mills D., Peralta J.M., Fine A.H. (2021). Working with Companion Animals, and Especially Dogs, in Therapeutic and Other AAI Settings. The Welfare of Animals in Animal-Assisted Interventions: Foundations and Best Practice Methods.

[12] Gee N.R., Rodriguez K.E., Fine A.H., Trammell J.P. (2021). Dogs Supporting Human Health and Well-Being: A Biopsychosocial Approach. Front. Vet. Sci..

[13] Stergiou A.N., Mattila-Rautiainen S., Varvarousis D.N., Tzoufi M., Plyta P., Beris A., Ploumis A. (2023). The Efficacy of Equine Assisted Therapy Intervention in Gross Motor Function, Performance, and Spasticity in Children with Cerebral Palsy. Front. Vet. Sci..

[14] Heussen N., Häusler M. (2022). Equine-Assisted Therapies for Children with Cerebral Palsy: A Meta-Analysis. Pediatrics.

[15] Steff K., Grasemann M., Ostermann K., Goretzki S.C., Rath P.-M., Reinhardt D., Schündeln M.M. (2024). Feasibility, Efficacy, and Safety of Animal-Assisted Activities with Visiting Dogs in Inpatient Pediatric Oncology. World J. Pediatr..

[16] Sobo E.J., Eng B., Kassity-Krich N. (2006). Canine Visitation (Pet) Therapy: Pilot Data on Decreases in Child Pain Perception. J. Holist. Nurs..

[17] Calcaterra V., Veggiotti P., Palestrini C., De Giorgis V., Raschetti R., Tumminelli M., Mencherini S., Papotti F., Klersy C., Albertini R. (2015). Post-Operative Benefits of Animal-Assisted Therapy in Pediatric Surgery: A Randomised Study. PLoS ONE.

[18] Ávila-Álvarez A., Pardo-Vázquez J., De-Rosende-Celeiro I., Jácome-Feijoo R., Torres-Tobío G. (2020). Assessing the Outcomes of an Animal-Assisted Intervention in a Paediatric Day Hospital: Perceptions of Children and Parents. Animals.

[19] Wagner C.M., Butcher H.K., Clarke M.F. (2023). Nursing Interventions Classification (NIC).

[20] Zhang T., Wu X., Peng G., Zhang Q., Chen L., Cai Z., Ou H. (2021). Effectiveness of Standardized Nursing Terminologies for Nursing Practice and Healthcare Outcomes: A Systematic Review. Int. J. Nurs. Knowl..

[21] Rodríguez-Suárez C.-A., González-de la Torre H., Hernández-De Luis M.-N., Fernández-Gutiérrez D.-Á., Martínez-Alberto C.-E., Brito-Brito P.-R. (2023). Effectiveness of a Standardized Nursing Process Using NANDA International, Nursing Interventions Classification and Nursing Outcome Classification Terminologies: A Systematic Review. Healthcare.

[22] Bertocchi L., Dante A., La Cerra C., Masotta V., Marcotullio A., Jones D., Petrucci C., Lancia L. (2023). Impact of Standardized Nursing Terminologies on Patient and Organizational Outcomes: A Systematic Review and Meta-Analysis. J. Nurs. Scholarsh..

[23] McCarthy E., Guerin S. (2022). Family-Centred Care in Early Intervention: A Systematic Review of the Processes and Outcomes of Family-Centred Care and Impacting Factors. Child Care Health Dev..

[24] Gómez-Cantarino S., García-Valdivieso I., Moncunill-Martínez E., Yáñez-Araque B., Ugarte Gurrutxaga M.I. (2020). Developing a Family-Centered Care Model in the Neonatal Intensive Care Unit (NICU): A New Vision to Manage Healthcare. Int. J. Environ. Res. Public Health.

[25] Seniwati T., Rustina Y., Nurhaeni N., Wanda D. (2023). Patient and Family-Centered Care for Children: A Concept Analysis. Belitung Nurs. J..

[26] Lechner B.E., Kukora S.K., Hawes K. (2024). Equity, Inclusion and Cultural Humility: Contemporizing the Neonatal Intensive Care Unit Family-Centered Care Model. J. Perinatol..

[27] Kokorelias K.M., Gignac M.A.M., Naglie G., Cameron J.I. (2019). Towards a Universal Model of Family Centered Care: A Scoping Review. BMC Health Serv. Res..

[28] London M.D., Mackenzie L., Lovarini M., Dickson C., Alvarez-Campos A. (2020). Animal Assisted Therapy for Children and Adolescents with Autism Spectrum Disorder: Parent Perspectives. J. Autism Dev. Disord..

[29] O’Brien B.C., Harris I.B., Beckman T.J., Reed D.A., Cook D.A. (2014). Standards for Reporting Qualitative Research: A Synthesis of Recommendations. Acad. Med..

[30] Tufford L., Newman P. (2012). Bracketing in Qualitative Research. Qual. Social Work..

[31] Eatough V., Smith J.A., Willig C., Stainton-Rogers W. (2017). Interpretative Phenomenological Analysis. Qualitative Research in Psychology.

[32] Smith J.A., Larkin M., Flowers P. (2021). Interpretative Phenomenological Analysis: Theory, Method and Research.

[33] Cohen M., Kahn D., Steeves R. (2000). Hermeneutic Phenomenological Research: A Practical Guide for Nurse Researchers.

[34] Streubert H., Carpenter D.R. (2011). Qualitative Research in Nursing: Advancing the Humanistic Imperative.

[35] World Medical Association (2013). World Medical Association Declaration of Helsinki: Ethical Principles for Medical Research Involving Human Subjects. JAMA.

[36] Hinic K., Kowalski M.O., Holtzman K., Mobus K. (2019). The Effect of a Pet Therapy and Comparison Intervention on Anxiety in Hospitalized Children. J. Pediatr. Nurs..

[37] Vagnoli L., Caprilli S., Vernucci C., Zagni S., Mugnai F., Messeri A. (2015). Can Presence of a Dog Reduce Pain and Distress in Children during Venipuncture?. Pain Manag. Nurs..

[38] Berry A., Borgi M., Francia N., Alleva E., Cirulli F. (2013). Use of Assistance and Therapy Dogs for Children with Autism Spectrum Disorders: A Critical Review of the Current Evidence. J. Altern. Complement. Med..

[39] Leighton S.C., Rodriguez K.E., Nieforth L.O., O’Haire M.E. (2023). Service Dogs for Autistic Children and Family System Functioning: A Constant Comparative Analysis. Front. Psychiatry.

[40] Beetz A., Uvnäs-Moberg K., Julius H., Kotrschal K. (2012). Psychosocial and Psychophysiological Effects of Human-Animal Interactions: The Possible Role of Oxytocin. Front. Psychol..

[41] Busch C., Tucha L., Talarovicova A., Fuermaier A.B.M., Lewis-Evans B., Tucha O. (2016). Animal-Assisted Interventions for Children with Attention Deficit/Hyperactivity Disorder: A Theoretical Review and Consideration of Future Research Directions. Psychol. Rep..

[42] Nap-van der Vlist M.M., van der Wal R.C., Grosfeld E., van de Putte E.M., Dalmeijer G.W., Grootenhuis M.A., van der Ent C.K., van den Heuvel-Eibrink M.M., Swart J.F., Bodenmann G. (2021). Parent-Child Dyadic Coping and Quality of Life in Chronically Diseased Children. Front. Psychol..

[43] Kerr M.L., Rasmussen H.F., Smiley P.A., Buttitta K.V., Borelli J.L. (2021). The Development of Toddlers’ Emotion Regulation within the Family System: Associations with Observed Parent-Child Synchrony and Interparental Relationship Satisfaction. Early Child. Res. Q..

[44] Stadelmann C., Senn M., Forster F., Rauch-Anderegg V., Nussbeck F.W., Johnson M.D., Iwanski A., Zimmermann P., Bodenmann G. (2023). Dyadic Coping Trajectories across the Transition to Parenthood: Associations with Child Mental Health Problems. J. Fam. Psychol..

[45] McCullough A., Ruehrdanz A., Jenkins M.A., Gilmer M.J., Olson J., Pawar A., Holley L., Sierra-Rivera S., Linder D.E., Pichette D. (2018). Measuring the Effects of an Animal-Assisted Intervention for Pediatric Oncology Patients and Their Parents: A Multisite Randomized Controlled Trial [Formula: See Text]. J. Pediatr. Oncol. Nurs..

[46] Silva N.B., Osório F.L. (2018). Impact of an Animal-Assisted Therapy Programme on Physiological and Psychosocial Variables of Paediatric Oncology Patients. PLoS ONE.

[47] Mahoney A.B., Akard T.F., Cowfer B.A., Dietrich M.S., Newton J.L., Gilmer M.J. (2024). Impact of Animal-Assisted Interaction on Anxiety in Children with Advanced Cancer and Their Caregivers. J. Palliat. Med..

[48] Gomes da Silva F., de Paula D.D., Alves L.M., Santos J.N. (2025). Benefits of Horseback Riding for Neurotypical Children and Adolescents: A Scoping Review. Codas.

[49] Peterson A. (2008). Donna J. Haraway, When Species Meet. J. Agric. Environ. Ethics.

[50] Bisht A., Walton S., Chandrasekaran S., Garner O. (2020). Straight from the Mini-Horse’s Mouth: Potential Carrier for Opportunistic Human Pathogens. Am. J. Infect. Control.

[51] Reddekopp J., Dell C.A., Rohr B., Fornssler B., Gibson M., Carey B., Stempien J. (2020). Patient Opinion of Visiting Therapy Dogs in a Hospital Emergency Department. Int. J. Environ. Res. Public Health.

[52] Grover S., Fitzpatrick A., Azim F.T., Ariza-Vega P., Bellwood P., Burns J., Burton E., Fleig L., Clemson L., Hoppmann C.A. (2022). Defining and Implementing Patient-Centered Care: An Umbrella Review. Patient Educ. Couns..

[53] Jeppesen E., Schmidt A.A., Skjødt C.K., Hybschmann J., Gjærde L.K., Thestrup J., Hansson H., Sørensen J.L. (2024). Educational Programmes for Paediatric Healthcare Professionals in Patient- and Family-Centred Care. A Scoping Review. Eur. J. Pediatr..

[54] Barchas D., Melaragni M., Abrahim H., Barchas E. (2020). The Best Medicine: Personal Pets and Therapy Animals in the Hospital Setting. Crit. Care Nurs. Clin. N. Am..

[55] Park M., Giap T.-T.-T., Lee M., Jeong H., Jeong M., Go Y. (2018). Patient- and Family-Centered Care Interventions for Improving the Quality of Health Care: A Review of Systematic Reviews. Int. J. Nurs. Stud..

